# Ageism against Older Adults: How do Intersecting Identities Influence Perceptions of Ageist Behaviors?

**DOI:** 10.1177/07334648231161937

**Published:** 2023-03-13

**Authors:** Hannah M. Gans, Michelle Horhota, Alison L. Chasteen

**Affiliations:** 1 Department of Psychology, 7938 University of Toronto - St George Campus, Toronto, ON, CA; 2 Department of Psychology, 3628 Furman University, Greenville, SC, USA

**Keywords:** ageism, perception, gender, race

## Abstract

Most ageism research has focused on prejudice against older people without considering their multiple intersecting identities. We investigated perceptions of ageist acts that targeted older individuals with intersecting racial (Black/White) and gender identities (men/women). Both young (18–29) and older (65+) adult Americans evaluated the acceptability of a variety of instances of hostile and benevolent ageism. Replicating prior work, benevolent ageism was seen as more acceptable compared to hostile ageism, and young adults rated ageist acts as more acceptable than older adults. Small intersectional identity effects were observed such that young adult participants perceived older White men to be the most acceptable targets of hostile ageism. Our research suggests that ageism is viewed differently depending on the age of the perceiver and the type of behavior exhibited. These findings also suggest intersectional memberships should be considered, but further research is needed given the relatively small effect sizes.

**What this paper adds**

• Across a variety of behaviors, benevolent ageist acts are consistently perceived as being more acceptable than hostile ageist acts, thus demonstrating the robust nature of this effect across current and past studies.
• Young adults’ acceptability ratings for hostile ageism and older adults’ acceptability ratings for benevolent ageism differ depending on the older target person’s intersecting identities.
• Young adult participants rated older White men as the most acceptable targets of hostile ageism compared to older Black men and women and older White women.

**Applications of study findings**

• Consider intersectionality with older adults in practice, policy, and research, as older adults’ race and gender identities result in different experiences for older people.
• Clinicians and practitioners should be aware of how benevolent and hostile ageism can take many different forms when working with older clients from varied backgrounds.

## Introduction

The demography of the American population is changing significantly, marked not only by an increase in the number of older adults, but also in the number of older adults that identify as racial minorities (United States Census Bureau, 2021). Specifically, the 65-and-over population in America is projected to double in size by 2060 to almost a quarter of the entire population (United States Census Bureau, 2021). Furthermore, by 2040, it is projected that the racial and ethnic minority composition of those 65 and older will increase by 115%, with the number of older Hispanic adults increasing by 161%, older African American adults increasing by 80%, older American Indian and Alaska Native adults increasing by 67%, and older Asian Americans increasing by 102% (United States Department of Health and Human Services, 2021).

Despite these changes in the older adult population, research on ageism toward older adults has been limited by its focus on age as the primary identity that older people hold (Burn et al., 2019; Chasteen et al., 2021a, 2021b, 2022; Rothermund & Brandtstädter, 2003; Van Borm et al., 2021). As a result, we know very little about how ageism is manifested towards older men and women of color. Previous literature has demonstrated the influence that intersectional identities have on person perception, and specifically, the way in which a target’s racial and gender identities intersect to form unique perceptions and stereotypes (Freeman & Ambady, 2011; Ghavami & Peplau, 2013; Hall et al., 2019; Kang & Chasteen, 2009, 2014; Mair, 2010; Martin et al., 2019; Neel & Lassetter, 2019; Schug et al., 2015). Therefore, it is important to broaden research on ageism to include older adults with varying racial and gender identities. The present paper seeks to expand current research on ageism by examining how ageism is applied towards older adults with intersecting racial (Black/White) and gender (men/women) identities.

### Ageism and its Associated Consequences

Ageism is defined as prejudice based on one’s age (Butler, 1969), manifested either as benevolent or hostile attitudes toward others (Cary et al., 2017). Benevolent ageism, rooted in the stereotype that older adults are warm but incompetent (Fiske et al., 2002), often results in patronizing behavior, including overaccommodative helping (Cary et al., 2017). Experiences of benevolent ageism are common among older adults (Chasteen et al., 2021a). Contrasting this, hostile ageism, rooted in the stereotype that older adults are neither warm nor competent, often manifests as contemptuous prejudice (Cuddy et al., 2008; Fiske et al., 2002), in which older adults may be neglected or abused (Cary et al., 2017).

Though manifested differently, both forms of ageism can adversely affect the mental and physical health of older adults (Chrisler et al., 2016; Dionigi, 2015; Marchiondo et al., 2019; Sargent-Cox et al., 2012). Thus, ageism research is critical; however, it has become increasingly important in recent years due to the rise in ageism associated with the COVID-19 pandemic (Ayalon et al., 2020; Derrer-Merk et al., 2022; Fraser et al., 2020; Spaccatini et al., 2022). More specifically, throughout the pandemic, older adults have been repeatedly portrayed as the most vulnerable and susceptible population, resulting in an increase in intergenerational tension as young adults began to blame older adults for precautionary public health measures (Ayalon, 2020; Derrer-Merk et al., 2022; Fraser et al., 2020; Spaccatini et al., 2022; Swift & Chasteen, 2021). Stereotype Embodiment Theory (SET, Levy, 2009), which argues that exposure to ageist stereotypes from a young age can result in the internationalization of these stereotypes which subsequently influence one’s own experiences of aging, suggests that the negative consequences from this intergenerational tension are twofold, whereby both older and young adults are susceptible to internalizing these negative stereotypes and thus potentially harming their current (older adults) and future (young adults) aging experiences. Thus, given the exacerbation of ageism during the pandemic, it is even more crucial than ever to understand how ageism operates so that we may move towards identifying ways to reduce it.

One method that has been shown to be useful in identifying how ageism is applied to older adults is to examine people’s acceptability ratings of ageist acts. Horhota et al. (2019) found that acceptability ratings of ageism can differ based on the type of ageism, the participant’s age, and the familiarity of the perpetrator to the older target. More specifically, they concluded that benevolent ageism is perceived as more acceptable than hostile ageism, older adults view hostile ageism as less acceptable compared with young adults, and the more familiar the perpetrator is to the older target, the more acceptable the ageist act is seen to be (Horhota et al., 2019). Although the findings of Horhota et al. (2019) provided an important first step in understanding the norms surrounding perceptions of ageist acts, their study did not consider whether such perceptions would vary depending on the older target person’s intersecting identities. With the rise of an older adult population that identifies as racial minorities (United States Census Bureau, 2021), it is important to expand this research to include older adults with diverse identities.

### Ageism and Intersectionality

Numerous studies have demonstrated the importance of considering a target’s intersecting identities in person perception, as each individual identity can lead to unique associated stereotypes (Freeman & Ambady, 2011; Hall et al., 2019; Neel & Lassetter, 2019). For example, in two separate studies, Ghavami and Peplau (2013) and Schug et al., (2015) demonstrated how a target’s racial and gender identities intersect to form unique perceptions and stereotypes. However, despite the plethora of theoretical models that have detailed how person perception is an interactive process, an individual’s age identity has often been overlooked (Freeman & Ambady, 2011; Hall et al., 2019; Neel & Lassetter, 2019).

In the few intersectionality research studies that have included age as an intersecting identity with race, they have focused mainly on men rather than women as the targets (e.g., Hurd Clarke & Korotchenko, 2016; Kang & Chasteen, 2009, 2014). For example, in a facial emotion change-detection study, participants evaluated emotional expressions on the faces of young and older Black and White men (Kang & Chasteen, 2009). Although the findings suggested that intersections of age and race do influence perceptions of negative emotions like anger on men’s faces, it remains unknown whether such results would generalize to women’s faces. Given that previous research has demonstrated that gender and race affect person perception (Kite et al., 2005; Martin et al., 2019; Walker & Melton, 2015), it is important to examine whether intersecting identities may also influence perceptions of older adults.

## Present Study

Despite a long history of research on ageism (e.g., Ayalon and Tesch-Römer, 2018; Butler, 1969; Kite et al., 2005; Nelson, 2002, 2016), surprisingly few studies have studied ageism towards older adults with diverse racial and gender identities (Bergeron & Lagacé, 2021; Chasteen et al., 2022; Koenig, 2018). Given this gap, in the present study, we examine how older individuals’ intersecting racial (Black/White) and gender (male/female) identities influence how acts of ageism against them are perceived. We extend the acceptability ratings paradigm used by Horhota et al., (2019), in which young and older participants rated the acceptability of a variety of benevolent and hostile ageist acts toward older people. In this study, we include both young adult and older adult participants and test whether ratings of the acceptability of benevolent and hostile ageist acts will vary as a function of the intersecting racial (Black/White) and gender (male/female) identities held by the older adult targets. Based on previous literature, we expected a main effect of participant age such that young adults will rate both hostile and benevolent ageist acts as more acceptable compared with older adults, regardless of the intersecting identities of the target (Horhota et al., 2019). We also expected a main effect of ageism type, in line with previous research, such that benevolent ageism would be rated as more acceptable compared to hostile ageism, regardless of the participant’s age and the target’s intersecting identities. (Horhota et al., 2019; North & Fiske, 2013). Lastly, we expected an intersectionality effect, such that a target’s intersecting racial and gender identities would lead to unique acceptability ratings (Freeman & Ambady, 2011; Ghavami & Peplau, 2013; Hall et al., 2019; Petsko & Bodenhausen, 2020); however, we had competing hypotheses about the direction of the intersectional effects. Our two primary competing accounts were grounded in the Intersectional Escape Hypothesis (Martin et al., 2019) and the Double Jeopardy Hypothesis (Beale, 1970; Blakemore & Boneham, 1994).

Specifically, the Intersectional Escape Hypothesis (Martin et al., 2019) suggests that older adults who are not viewed as prototypical of their group might escape age stereotypes and prejudice associated with the group. In this instance, compared with older White men, older White women and older Black adults will be seen as less acceptable targets, particularly for benevolent ageism, the more commonly experienced form.

Alternatively, the Double Jeopardy hypothesis (Beale, 1970; Blakemore & Boneham, 1994) predicts that those with multiple subordinate and stigmatized identities will be perceived as the most acceptable targets of the more blatant form of ageism, hostile ageism. Following this hypothesis, older White men would be perceived as the least acceptable targets, compared to older women and older Black individuals, all of whom have multiple subordinate, stigmatized identities. More detailed discussion about our pre-registered competing predictions can be viewed on the Open Science Framework:https://osf.io/kypcq/?view_only=bfcf89725bb74b76b0851da186ad628d.

## Methods

### Participants and Design

Given our four independent variables of participant age (young adult or older adult), ageism type (benevolent or hostile ageism), target gender (male or female), and target race (Black or White), we performed an a priori simulated power analysis using R 4.0.5 (R Core Team, 2021), specifying a small effect size (f = 0.14) and power of 0.90, resulting in a recommended sample of 226 participants (YA: *n* = 113; OA: *n* = 113), which would allow for 452 observations of acceptability ratings. To offset potential data loss, we oversampled for the study, recruiting 270 participants from the online platform Prolific in April 2022, and compensated them $1.30 USD. After removing duplicate and nonattentive responses, the final sample included 126 young adults and 131 older adults for a total of 257 participants and 514 observations of acceptability ratings (see Table 1 for demographic information). This study was approved by the university ethics board.

**Table 1.** Participant Demographics.

Demographic Characteristics	Young Adult Participants (*n* = 126)	Older Adult Participants (*n* = 131)
Participant age		
Mean (SD)	23.89 (3)	69.54 (4.81)
Participant gender		
Female	84 (66.7%)	66 (50.4%)
Male	39 (31.0%)	65 (49.6%)
Other	3 (5.4%)	NA
Participant race		
White	60 (47.6%)	123 (93.9%)
East/Southeast Asian	23 (18.3%)	2 (1.5%)
Latin/South American	17 (13.5%)	NA
Black	9 (7.1%)	3 (2.3%)
Multiracial	7 (5.6%)	NA
South Asian	5 (4.0%)	NA
Middle Eastern	4 (3.2%)	1 (0.8%)
Other	1 (0.8%)	NA
Participant education		
Some high school	2 (1.6%)	1 (0.8%)
High school/GED	11 (8.7%)	11 (8.4%)
Some college/University	43 (34.1%)	25 (19.1%)
College/Associate’s	8 (6.3%)	6 (4.6%)
Bachelor’s	54 (42.9%)	47 (35.9%)
Master’s	6 (4.8%)	33 (25.2%)
Doctoral/Professional	2 (1.6%)	8 (6.1%)

*Note.* A 7-point scale measuring education level indicated that older adults were moderately more educated compared with younger adults (M_OA_ = 4.66, M_YA_ = 4.00, t(252) = −3.87, *p* < 0.05).

### Procedure

Participants completed a 30-minute online Qualtrics survey at their own pace in their homes. Participants provided informed consent and were then randomly assigned to complete a measure of the acceptability of ageist acts (Horhota et al., 2019) for two of the following four identities: old White man, old White woman, old Black man, or old Black woman. Participants completed the entire scale first with reference to one of the two intersecting identities they were randomly assigned to, and then repeated the scale with reference to the other intersecting identity. The acceptability measure asked participants to rate the acceptability of benevolent and hostile ageist acts. The items were presented in a random order throughout. An example of a benevolent item is “Talking slower to an older _____ because it may take a while for them to understand things that are said to them.” An example of a hostile ageist item is “Avoiding having conversations with an older ____.” After rating the acceptability of each ageist act on a scale from 1 (Never Acceptable) to 5 (Always Acceptable) and completing a variety of exploratory measures that were for a separate project, participants completed a demographics form, were debriefed about the purpose of the study, and were compensated.

### Analysis Plan

All analyses for this study were performed using R 4.0.5 (R Core Team, 2021). We adapted items from the ageist acts used in Horhota et al., (2019), which originally included 13 benevolent ageist acts and 17 hostile ageist acts. However, four hostile ageist acts were removed as they were skewed and could not be transformed to reflect normality. Therefore, our measure included 13 benevolent ageist acts (α = 0.891) and 13 hostile ageist acts (α = 0.894), for a total of 26 ageist acts (α = 0.932).

Mean acceptability ratings for the hostile and benevolent items were calculated separately for each intersectional target. As participants evaluated more than one intersecting identity, we analyzed identity differences through a series of multilevel models with observations nested within participants, specifying random intercepts. Based on the model parameters, estimated marginal means were calculated and a priori pairwise comparisons were made to determine which identities had significantly different acceptability ratings from each other. The Benjamini–Hochberg Procedure was used to adjust the *p*-values to offset the potential for Type I errors.

As part of our long-term research plan, we were also interested in what intersectional identity effects might be occurring at the item level, so we ran secondary item-level analyses on each benevolent and hostile ageist act across young and older adult participants. The Benjamini–Hochberg Procedure was again used to adjust the *p*-values to offset the potential for Type I errors. Data and R script are available on the Open Science Framework: https://osf.io/j5y3z/?view_only=2809fb664ced4d3e8a3c73d020acdbba.

## Results

Below, we first report the analyses for the hostile and benevolent ageism composites, followed by the item-level analyses.

### Ageism Composite-Level Analyses

The multilevel model revealed two large main effects as significant. Consistent with our hypotheses, there was a main effect of age of participant (*F* (1, 254.6) = 30.36, *p* < 0.001, *d* = 0.70), and a main effect of type of ageism (*F* (1, 13092.8) = 2165.47, *p* < 0.001, *d* = 0.81). More specifically, ageist acts were rated as more acceptable by young adult participants (*M* = 2.77, *SE* = 0.06) compared to older adult participants (*M* = 2.34, *SE* = 0.05), and benevolent ageist acts were perceived as more acceptable (*M* = 2.99, *SE* = 0.04) compared to hostile ageist acts (*M* = 2.12, *SE* = 0.04). Two small main effects of target race (*F* (1, 13318.9) = 7.73, *p* < 0.01, *d* = 0.05), and target gender (*F* (1, 13318.9) = 4.92, *p* < 0.05, *d* = 0.04) were also revealed. However, it is important to note that the effect sizes are small, especially when considering the large sample size of this study, thus implying that ageist acts against older Black and White adults as well as ageist acts against older men and women are likely perceived as equally acceptable.

There were also three significant two-way interactions, including a target race by target gender interaction (*F* (1, 13112.3) = 3.87, *p* < 0.05, *d* = 0.04; see Table 2). In line with the Intersectional Escape Hypothesis, older White men were rated as the most acceptable target of ageism compared to older Black men/women and older White women. There was also a participant age by type of ageism interaction (*F* (1, 13092.8) = 12.45, *p* < 0.001, *d* = 0.06; see Table 2), such that the differences between young and older adults' acceptability ratings of benevolent ageism were larger than for hostile ageism. Finally, there was a type of ageism by target gender interaction (*F* (1, 13092.8) = 15.03, *p* < 0.001, *d* = 0.07; see Table 2), such that acceptability ratings of hostile ageism differed for older men and women targets but not for benevolent ageism.

**Table 2.** Two-Way Interactions.

Independent Variable		Type of Ageism (A)	
Participant age		Hostile	Benevolent
Young	M (SE)	2.30 (0.06)_a_	3.23 (0.06)_c_
Old	M (SE)	1.94 (0.06)_b_	2.74 (0.06)_d_
Target gender			
Male	M (SE)	2.18 (0.04)_e_	2.97 (0.04)_g_
Female	M (SE)	2.06 (0.04)_f_	3.00 (0.04)_g_
		Target gender (B)	
Target race		Male	Female
White	M (SE)	2.63 (0.04)_h_	2.54 (0.04)_i_
Black	M (SE)	2.52 (0.04)_i_	2.52 (0.04)_i_

*Note.* Higher scores represent an increased perception that the individual is an acceptable target of ageism. Scales range from 1 to 5.

Means that share a subscript are not significantly different from each other, *p*s < .05, whereas means that do not share a subscript are significantly different from each other, *ps* > .05. See results section for exact *p*-values.

**Figure 1.**
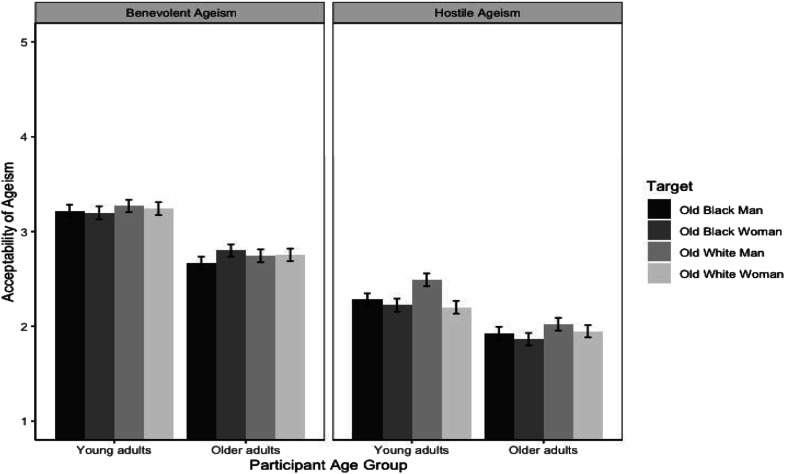
Acceptability of ageism as a function of type of ageism, target race, target gender, and participant age group.

Analyses of the benevolent and hostile ageism composites also revealed a four-way interaction between ageism type, target race, target gender, and participant age (*F* (1, 13092.8) = 4.98, *p* < 0.05, *d* = 0.04; see Figure 1). Pairwise comparisons revealed support for the Intersectional Escape Hypothesis among the young adult participants, who rated older White men as the most acceptable target of hostile ageism (*M* = 2.49, *SE* = .07), compared to all other intersectional targets, who were all rated as equally acceptable (*M*_*OWW*_ = 2.20; *M*_*OBW*_ = 2.22; *M*_*OBM*_ = 2.28, all *SE*s = 0.07). In contrast, older adults did not rate the intersectional targets differently (*M*_*OWM*_ = 2.02; *M*_*OWW*_ = 1.95; *M*_*OBW*_ = 1.86; *M*_*OBM*_ = 1.93, all *SE*s = 0.07).

### Ageism Item-Level Analyses

Next, we conducted our secondary analyses using a multilevel model to investigate potential intersectionality effects at the item level to determine whether the acceptability of certain ageist acts vary more depending on the intersecting identities of the older target. We found that several items displayed significant intersectional variability in acceptability ratings (see Tables 1 and S2 in Supplementary Materials). Specifically, in terms of benevolent ageist acts, offering to help a Black or White woman across the street was perceived as more acceptable (*p* < 0.05) compared with helping a Black or White man. These differences were driven by older adults’ ratings, whereas young adults did not demonstrate intersectional variability in their ratings for benevolent ageist acts. With regards to hostile ageist acts, most of the intersectional variability in acceptability ratings was driven by young participants’ ratings. Specifically, for young adult participants, older White men were rated as more acceptable targets of ageist humor and ageist exclusion (e.g., ignoring older White men) than any other intersectional targets. These intersectional differences were not seen in older adult participants’ ratings.

## Discussion

The current study investigated age differences in perceptions of the acceptability of hostile and benevolent ageist behaviors directed at older targets with intersecting racial (Black/White) and gender (male/female) identities. Specifically, participants rated how acceptable a variety of benevolent and hostile ageist acts were when directed towards older White men, older Black men, older White women, or older Black women. Composite scores of acceptability ratings for benevolent and hostile ageist acts were analyzed, and following this, item-level analyses were conducted.

Our analyses demonstrated patterns similar to those observed by Horhota et al., (2019) and supported our pre-registered hypotheses, such that acceptability ratings of ageism varied according to the type (benevolent vs. hostile ageism) as well as the participant’s age (young vs. older adults). More specifically, benevolent ageism was more readily accepted, regardless of the participant’s age or the target’s intersecting identities, suggesting that it is often perceived as polite and helpful, and not harmful or invalidating.

Further, in line with our hypotheses, young adult participants rated both hostile and benevolent ageism as more acceptable than older adult participants, regardless of the targets’ intersecting identities, suggesting possible ingroup-outgroup differences. These differences may be the result of young adult participants identifying less with the older adult targets compared with older adult participants who see themselves within the target age category. Another reason for these participant age differences may be explained by the fact that young adults hold more negative stereotypes about older adults than other age groups (Rupp et al., 2005), thus resulting in higher acceptability ratings, specifically with regards to hostile ageism. Notably, these patterns concerning the acceptability of benevolent versus hostile ageism and regarding participant age seem to be remarkably stable, given that data collection for the present study took place in a different sample approximately 5 years after data collected by earlier work (Horhota et al., 2019) and during a time of increasing ageism as a result of the COVID-19 pandemic (Ayalon, 2020).

Unique to this study were the results for older targets with intersecting identities. Our analyses for the benevolent and hostile ageism composites revealed that, contrary to our predictions for hostile ageism regarding The Double Jeopardy Hypothesis, older White men were perceived to be the most acceptable targets of hostile ageism, particularly by young adult participants, compared to the other targets who were rated equally. Furthermore, our analyses of the individual items demonstrated that these higher acceptability ratings were primarily driven by older White men being viewed as more acceptable targets of ageist humor and ageist exclusion. As such, these results seem to be more aligned with the Intersectional Escape Hypothesis (Martin et al., 2019), in that White women and older Black individuals were perceived as the least acceptable targets of ageism.

Another factor that could explain why older White women and older Black individuals were seen as less acceptable targets could relate to recent increases in social justice advocacy supporting anti-racism and anti-sexism but a continuous endorsement of succession-based ageism (Martin & North, 2022). Social justice movements including feminist movements and racial equality movements (i.e., #BlackLivesMatter) have gained significant momentum in the last few years (Buchanan et al., 2020; Hartocollis & Alcindor, 2017). Because of this, there has been a heightened awareness of bias, which may have prompted young adults to perceive the least marginalized group (i.e., old White men) to be the most acceptable targets of hostile ageist behavior compared to more marginalized groups (i.e., Black older adults and women).

It is also possible that the higher ageism acceptability ratings occurred for older White men because of perceived violations of prescriptive age stereotypes. North and Fiske (2012, 2013) suggest that intergenerational tensions may occur when older adults are seen to be violating prescriptive age stereotypes regarding domains such as envied resource succession, leading to hostile behavior among young adults towards older adults who have violated this prescriptive stereotype (North & Fiske, 2012, 2013). Moreover, Martin et al. (2019) found that older men are seen as more of a resource threat than older women. Therefore, older White men might elicit a sense of hostility among young adult participants if they are perceived as being the faces of power in society (i.e., violating the prescriptive stereotype of resource succession; Lu et al., 2020; Zweigenhaft, 2020) relative to other, more marginalized older adults (i.e., Black targets and women).

### Strengths and Limitations

The current work presents a pre-registered study featuring a mixed-model design and replication of effects from previous research (Horhota et al., 2019). The data also shed light on an under-researched question relevant to older adults with intersecting identities, revealing that older adults are perceived differently based on the intersection of their racial and gender identities. The implications of this are significant for both service providers working with older adults as well as activists and researchers advocating for older people. Healthcare providers and service providers, for example, should be aware of the different experiences older adults with intersecting identities may have, especially because any ageist attitudes older adults are exposed to may be internalized (Levy et al., 2020; 2009), resulting in their treatment being less effective.

In addition, this study highlights the fact that benevolent ageism is regarded as acceptable towards targets with various intersectional identities, and by perceivers of various ages, suggesting the need for further work on how to reduce the perceived acceptability of benevolent ageism. Previous literature has suggested that by moderately confronting a benevolent ageist comment, older adults can avoid enduring social penalties, while reducing the perceived appropriateness of the ageist behavior (Chasteen et al., 2021b), however, additional research that includes intersectional targets is needed.

The present study was limited to examining two races (White, Black) and two genders (male, female) as intersecting identities, and the intersectional effects were relatively small. Therefore, future research should aim to replicate the current results while also investigating the potential intersectional effects of other racial and gender identities. More specifically, because the relative proportion of Hispanic, American Indian, Alaska Native, and Asian American older adults is increasing (United States Department of Health and Human Services, 2021), and the number of adults who identify as non-binary is increasing (Barajas, 2021), future studies should seek to broaden the scope of the racial and gender identities that are examined. Finally, future research should seek to better understand how intersections between age and other identities are accounted for by theoretical models of intersectionality. Only by doing so will we uncover individuals’ unique aging experiences.

Additionally, even though the ageist acts used in this study allowed us to tightly control our experiment and replicate previous results, the acts were described briefly and provided little information about the targets other than their racial and gender identities. Despite this approach to experimental control, it is possible that participants filled in gaps with their own assumptions about the targets, which may have influenced their responses. Future research should examine the effects of providing more context about older intersectional targets on perceptions of ageist acts.

Finally, the samples reported are convenience samples recruited from an online platform in which the majority of participants were White, educated, and had access and familiarity with the Internet and electronic devices. Thus, they are not representative of the broader population and caution should be exercised before generalizing the results. Future research may wish to purposefully sample groups of interest that are more diverse in terms of racial identities and educational achievements, as well as those who have less familiarity and access to the Internet and electronic devices.

## Conclusion

In conclusion, this study contributes valuable insights into how ageism towards intersectional targets is perceived. As such, these findings can be used to develop interventions to reduce ageism expressed toward older adults from a variety of backgrounds and facilitate a more inclusive approach to optimizing the lives of older people.

## Supplemental Material

Supplemental Material - Ageism against Older Adults: How do Intersecting Identities Influence Perceptions of Ageist Behaviors? Supplemental Material for Ageism against Older Adults: How do Intersecting Identities Influence Perceptions of Ageist Behaviors? by Hannah M. Gans, Michelle Horhota, and Alison L. Chasteen in Journal of Applied Gerontology
