# Supplementary material for: Ageism against Older Adults: How do Intersecting Identities Influence Perceptions of Ageist Behaviors?
Source: J Appl Gerontol. 2023 Mar 13;42(6):1191–9. doi: 10.1177/07334648231161937 (PMC10201080; doi:10.1177/07334648231161937)
Supplement: Supplemental Material - Ageism against Older Adults: How do Intersecting Identities Influence Perceptions of Ageist Behaviors? [file sj-pdf-1-jag-10.1177_07334648231161937.pdf]

S1. A) Item level differences in young adult participants' ratings of hostile ageist acts.

Ageist Act	Old White Man	Old Black Man	Old White Woman	Old Black Woman
**Sending birthday cards to an ____ that jokes about their age.*	*3.14 (0.12)$_{ab}$*	*3.17 (0.12)$_{cd}$*	*2.63 (0.13)$_{ac}$*	*2.48 (0.13)$_{bd}$*
Avoiding having conversations with an ____	*2.39 (0.12)$_{ab}$*	*1.76 (0.11)$_{a}$*	*2.10 (0.12)*	*1.77 (0.12)$_{b}$*
**Telling an ____ jokes about old age.*	*2.81 (0.11)$_{ab}$*	*2.73 (0.11)*	*2.33 (0.12)$_{a}$*	*2.31 (0.12)$_{b}$*
When an ____ can't remember something, saying, ''That's what they call a 'Senior Moment'.''	*2.30 (0.12)*	*2.10 (0.12)*	*1.99 (0.12)*	*2.10 (0.13)*
Talking louder to an ____ because of their age.	*3.04 (0.12)*	*2.77 (0.12)*	*2.81 (0.12)*	*2.81 (0.13)*
Ignoring an ____ because of their age.	*1.63 (0.10)*	*1.47 (0.10)*	*1.57 (0.10)*	*1.58 (0.10)*
**Avoiding an ____ because of their age.*	*2.19 (0.10)$_{a}$*	*1.74 (0.11)*	*1.96 (0.12)*	*1.64 (0.12)$_{a}$*
Calling an ____, ''young lady,'' or calling an____ man, ''young man.''	*2.77 (0.13)*	*2.76 (0.13)*	*2.79 (0.13)*	*2.96 (0.14)*
Telling a joke that pokes fun at an ____	*2.48 (0.11)$_{a}$*	*2.19 (0.11)*	*1.96 (0.11)$_{a}$*	*2.06 (0.12)*
**Perceiving an ____ as unattractive because of their age.*	*2.81 (0.12)$_{a}$*	*2.36 (0.12)*	*2.39 (0.13)*	*2.27 (0.13)$_{a}$*
Assuming that an ____ cannot hear well because of their age.	*2.50 (0.11)*	*2.42 (0.12)*	*2.47 (0.12)*	*2.35 (0.12)*
Assuming that an ____ cannot understand complex information because of their age.	*2.01 (0.11)*	*1.92 (0.10)*	*1.99 (0.11)*	*1.92 (0.11)*
Not allowing an ____ to participate in activities because of their age	*2.20 (0.11)*	*2.16 (0.11)*	*2.32 (0.11)*	*2.12 (0.12)*

*Note. Results are in the form of Mean (Standard Error). Items with an * had a significant Target Race x Target Gender interaction (Fs > 1.30, ps < 0.05). Means that share a subscript significantly differ from each other, ps < .05.*

S1. B) Item level differences in young adult participants' ratings of benevolent ageist acts.

Ageist Act	Old White Man	Old Black Man	Old White Woman	Old Black Woman
Complimenting an ____ on how well they look, despite their age	*3.63 (0.13)*	*3.56 (0.13)*	*3.44 (0.13)*	*3.37 (0.14)*
Holding doors open for an ____	*4.41 (0.09)*	*4.57 (0.09)*	*4.66 (0.09)*	*4.66 (0.1)*
Telling ____, ''You're too old for that'', so that they don't get hurt	*2.50 (0.11)*	*2.28 (0.11)*	*2.30 (0.11)*	*2.23 (0.12)*
Offering to help an ____ cross the street.	*4.13 (0.11)*	*4.14 (0.11)*	*4.28 (0.12)*	*4.30 (0.12)*
When finding out an ____'s age, saying, ''You don't look that old.''	*3.32 (0.13)*	*3.17 (0.12)*	*3.25 (0.13)*	*3.11 (0.13)*
When an ____ has an ailment, saying, ''That's normal at your age'', so as to not worry them.	*2.81 (0.11)*	*2.92 (0.11)*	*2.83 (0.11)*	*2.74 (0.12)*
Talking slower to ____ because it may take a while for them to understand things that are said to them.	*2.97 (0.12)*	*2.77 (0.12)*	*2.75 (0.13)*	*2.74 (0.13)*
Using simple words when talking to ____ so that they can understand more easily	*2.88 (0.12)*	*2.59 (0.12)*	*2.90 (0.12)*	*2.66 (0.13)*
Suggesting that an ___ take alternative forms of transportation rather than driving on their own so that they don't get stressed out by traffic.	*3.09 (0.12)*	*2.98 (0.12)*	*3.08 (0.13)*	*2.81 (0.13)*
Offering to carry an ___'s grocery bags to their car because of their age	*4.06 (0.11)*	*4.09 (0.11)*	*4.15 (0.11)*	*4.29 (0.12)*
Telling an ____ that you enjoyed conversing with him/her despite his/her age.	*2.77 (0.14)*	*2.84 (0.14)*	*2.67 (0.14)*	*2.60 (0.15)*

Shielding an ____ by not sharing information with them to avoid upsetting them	*2.85 (0.11)*	*2.70 (0.11)*	*2.85 (0.12)*	*2.65 (0.12)*
Repeating things to an ____ because they might not understand the information the first time	*3.45 (0.12)*	*3.00 (0.12)*	*3.21 (0.13)*	*2.99 (0.13)*

Note. Results are in the form of Mean (Standard Error).

S2. A) Item level differences in older adult participants' ratings of hostile ageist acts.

Ageist Act	Old White Man	Old Black Man	Old White Woman	Old Black Woman
Sending birthday cards to an ____ that jokes about their age.	*2.90 (0.14)*	*2.78 (0.14)*	*2.64 (0.14)*	*2.67 (0.14)*
Avoiding having conversations with an ____	*1.71 (0.10)*	*1.61 (0.10)*	*1.72 (0.10)*	*1.64 (0.10)*
Telling an ____ jokes about old age.	*2.28 (0.12)*	*2.25 (0.12)*	*2.07 (0.11)*	*2.07 (0.11)*
When an ____ can't remember something, saying, ''That's what they call a 'Senior Moment'.''	*2.38 (0.12)*	*2.24 (0.12)*	*2.27 (0.12)*	*2.33 (0.12)*
Talking louder to an ____ because of their age.	*2.29 (0.12)*	*2.09 (0.12)*	*2.08 (0.11)*	*2.01 (0.11)*
Ignoring an ____ because of their age.	*1.41 (0.08)*	*1.39 (0.08)*	*1.35 (0.08)*	*1.39 (0.08)*
Avoiding an ____ because of their age.	*1.59 (0.09)*	*1.50 (0.09)*	*1.52 (0.09)*	*1.60 (0.09)*
Calling an ____, ''young lady,'' or calling an ____, ''young man.''	*2.64 (0.13)*	*2.34 (0.13)*	*2.50 (0.13)*	*2.60 (0.13)*
Telling a joke that pokes fun at an ____	*1.92 (0.11)*	*1.72 (0.11)*	*1.66 (0.10)*	*1.41 (0.10)*
Perceiving an ____ as unattractive because of their age.	*1.97 (0.11)*	*1.68 (0.11)*	*1.85 (0.11)*	*1.71 (0.11)*
Assuming that an ____ cannot hear well because of their age.	*1.96 (0.11)*	*1.86 (0.11)*	*1.84 (0.11)*	*1.86 (0.11)*
Assuming that an ____ cannot understand complex information because of their age.	*1.59 (0.11)*	*1.73 (0.10)*	*1.63 (0.10)*	*1.57 (0.10)*
Not allowing an ____ to participate in activities because of their age	*1.73 (0.11)*	*1.75 (0.11)*	*1.88 (0.10)*	*1.74 (0.11)*

Note. Results are in the form of Mean (Standard Error).

S2. B) Item level differences in older adult participants' ratings of benevolent ageist acts.

Ageist Act	Old White Man	Old Black Man	Old White Woman	Old Black Woman
Complimenting an ____ on how well they look, despite their age	*3.02 (0.15)*	*3.09 (0.15)*	*3.10 (0.15)*	*3.22 (0.15)*
Holding doors open for an ____	*4.16 (0.11)*	*4.18 (0.11)*	*4.53 (0.11)*	*4.48 (0.11)*
Telling ____, "You're too old for that", so that they don't get hurt	*2.04 (0.11)*	*2.05 (0.11)*	*1.99 (0.11)*	*1.96 (0.11)*
**Offering to help an ____ cross the street.*	*3.51 (0.13)$_{ab}$*	*3.67 (0.13)*	*4.04 (0.12)$_{a}$*	*3.95 (0.12)$_{b}$*
When finding out an ____'s age, saying, "You don't look that old."	*3.15 (0.14)*	*2.90 (0.14)*	*2.99 (0.13)*	*3.14 (0.13)*
When an ____ has an ailment, saying, "That's normal at your age", so as to not worry them.	*2.23 (0.12)*	*2.19 (0.12)*	*2.25 (0.11)*	*2.29 (0.11)*
Talking slower to ____ because it may take a while for them to understand things that are said to them.	*2.12 (0.12)*	*2.03 (0.12)*	*2.06 (0.12)*	*2.05 (0.11)*
Using simple words when talking to ____ so that they can understand more easily	*2.21 (0.12)*	*2.05 (0.12)*	*2.20 (0.12)*	*2.05 (0.12)*
Suggesting that an ___ take alternative forms of transportation rather than driving on their own so that they don't get stressed out by traffic.	*2.56 (0.12)*	*2.30 (0.12)*	*2.31 (0.12)*	*2.40 (0.12)*
Offering to carry an ___'s grocery bags to their car because of their age	*3.72 (0.13)*	*3.65 (0.13)*	*4.03 (0.13)*	*3.86 (0.13)*
Telling an ____ that you enjoyed conversing with him/her despite his/her age.	*2.37 (0.15)*	*2.48 (0.15)*	*2.36 (0.14)*	*2.18 (0.15)*

Shielding an ____ by not sharing information with them to avoid upsetting them	*2.18 (0.11)*	*2.11 (0.11)*	*2.21 (0.11)*	*2.32 (0.11)*
Repeating things to an ____ because they might not understand the information the first time	*2.16 (0.13)*	*2.13 (0.13)*	*2.11 (0.12)*	*2.18 (0.12)*

*Note. Results are in the form of Mean (Standard Error). Items with an * had a significant Target Race x Target Gender interaction (Fs > 1.30, ps < 0.05). Means that share a subscript significantly differ from each other, ps < .05.*
